# Green Microfluidic Method for Sustainable and High-Speed Analysis of Basic Amino Acids in Nutritional Supplements

**DOI:** 10.3390/molecules29235554

**Published:** 2024-11-25

**Authors:** Iva Pukleš, Csilla Páger, Nikola Sakač, Bojan Šarkanj, Dean Marković, Marija Kraševac Sakač, Marija Jozanović

**Affiliations:** 1 Department of Chemistry, Josip Juraj Strossmayer University of Osijek, Cara Hadrijana 8, HR-31000 Osijek, Croatia; jcf9b6@pte.hu; 2 Doctoral School of Chemistry, University of Pécs, Ifjúság útja, 7624 Pécs, Hungary; 3 Department of Analytical and Environmental Chemistry, Faculty of Sciences, University of Pécs, Ifjúság útja, 7624 Pécs, Hungary; 4 Institute of Bioanalysis, Medical School, Szentágothai Research Center, University of Pécs, 7624 Pécs, Hungary; csilla.pager@aok.pte.hu; 5 Faculty of Geotechnical Engineering, University of Zagreb, Hallerova 7, HR-42000 Varaždin, Croatia; nsakac@gfv.unizg.hr (N.S.); or msakac@gfv.unizg.hr (M.K.S.); 6 Department of Food Technology, University North, Trg dr. Žarka Dolinara 1, HR-48000 Koprivnica, Croatia; bsarkanj@unin.hr; 7 Department of Biotechnology, University of Rijeka, Radmile Matejčić 2, HR-51000 Rijeka, Croatia; dean.markovic@biotech.uniri.hr; 8 Faculty of Chemical Engineering and Technology, University of Zagreb, Trg Marka Marulića 19, HR-10000 Zagreb, Croatia

**Keywords:** L-arginine, L-ornithine, L-lysine, amino acids, capacitively coupled contactless conductivity detection, nutritional supplements, microchip electrophoresis, sustainable

## Abstract

Amino acids (AAs) have broad nutritional, therapeutic, and medical significance and thus are one of the most common active ingredients of nutritional supplements. Analytical strategies for determining AAs are high-priced and often limited to methods that require modification of AA polarity or incorporation of an aromatic moiety. The aim of this work was to develop a new method for the determination of L-arginine, L-ornithine, and L-lysine on low-cost microchip electrophoresis instrumentation conjugated with capacitively coupled contactless conductivity detection. A solution consisting of 0.3 M acetic acid and 1 × 10^−5^ M iminodiacetic acid has been identified as the optimal background electrolyte, ensuring the shortest possible analysis time. The short migration times of amino acids (t ≤ 64 s) and method simplicity resulted in high analysis throughput with high precision and linearity (R^2^ ≥ 0.9971). The limit of detection values ranged from 0.15 to 0.19 × 10^−6^ M. The accuracy of the proposed method was confirmed by recovery measurements. The results were compared with CE-UV-VIS and HPLC-DAD methods and showed good agreement. This work represents the first successful demonstration of the ME-C^4^D analysis of L-arginine, L-ornithine, and L-lysine in real samples.

## 1. Introduction

Essential and non-essential amino acids (AAs) are crucial in sustaining optimal physiological processes and fundamental biological functions in the human body. Therefore, there is an increased requirement for adding AAs to nutritional supplements to improve certain physiological conditions, sustain good health, and for various therapeutic purposes. For instance, numerous data are available on the physiological importance of L-arginine, L-ornithine, and L-lysine; thus, these AAs are common active ingredients in different nutritional supplement formulations. L-arginine has an important role in the cardiovascular system [1,2], protein metabolism, immune response [3,4], and cell growth and regeneration [5]. Today, nutritional supplements containing L-arginine are most frequently used to improve sexual health [6,7]. Contributing to ammonia detoxification within the urea cycle, L-arginine is converted to another biologically valuable AA, L-ornithine [8]. Nutritional supplements containing L-ornithine are usually consumed to reduce physical exhaustion [9,10]. The supplement containing L-lysine is popular since it contributes to proper tissue and protein synthesis [11], absorption of calcium [12], and maintaining nitrogen balance [13]. Formulations containing L-lysine are used to treat herpes [14], sports injuries, post-operation recovery, and other medical conditions [15].

The growing consumer interest and medical demands for nutritional supplements containing AAs led to an increase in their production. As a result, it is necessary to implement regular and precisely defined quality control for commercial products to avoid potential health risks [16]. It is often debated which analytical method is the most suitable for supplement analysis since the reliable determination of AAs is usually performed by complex and high-priced instrumentations such as high-performance liquid chromatography (HPLC) with optical or mass spectrometry (MS) detectors [17]. The drawbacks of HPLC methods are the usage of high volumes of toxic solvents and sample derivatization. Listed drawbacks led to a trend where such products are often analyzed by analytical methods developed by the industries themselves, even though they have not been standardized or validated [18]. An overview of the available literature showed that HPLC is often replaced by capillary electrophoresis (CE) [19,20,21] as a more rapid and greener analytical technique usually used for the analysis of pharmaceuticals and biopharmaceuticals [22,23]. This is not surprising since nutritional supplements can be regarded as a grey zone between foods and pharmaceuticals.

Microchip electrophoresis (ME) devices have been demonstrated as powerful tools to analyze AAs. Moreover, their importance in analytical science has been established since they offer many advantages, such as low cost, rapid analysis, low consumption of samples and reagents, and effective separation. Compared to CE, the method optimization is more demanding due to the short separation path [24], while the small sample volume results in reduced sensitivity. Different approaches have been presented for enhancing sensitivity and separation efficiency in ME. Field-amplified stacking with reversed-field stacking was used by Wu et al. to enhance the sensitivity in ME-laser-induced fluorescence (LIF) analysis. The reported LOD of 2.5 × 10^−10^ M for L-lysine was the lowest reported in the literature for the ME analysis of lysine [25]. Lapos et al. presented a dual detection mode based on amperometric and LIF detection for the simultaneous analysis of a broad spectrum of analytes, including 4-chloro-7-nitrobenzofurazan-labeled L-arginine and catechol [26]. Wang et al. added Tween 20 in the background electrolyte (BGE) to enhance the separation efficiency of arginine, proline, histidine, and threonine in ME [27]. Another effective approach to improve separation efficiency is the modification of the microchip’s inner surface. Liang et al. used a polydopamine/gold nanoparticle-coated PDMS microchip to separate arginine, proline, histidine, valine, and threonine [28]. Qiu et al. reported a layer-by-layer assembly technique for coating PDMS microchannels with titanium dioxide nanoparticles [29]. In ME analysis, fluorescence detectors are mostly used since the derivatization of AAs facilitates sensitive detection. Voltammetric detection of L-arginine, L-ornithine, and L-lysine cannot be achieved by straightforward analysis since these AAs are not electroactive. To produce electrochemically detectable derivates, Wang et al. labeled AAs with NDA/cyanide [30]. Liang et al. employed slightly basic borate BGE in which the Cu electrodes were dissolved, forming an electroactive complex with AAs [31]. Capacitively coupled contactless conductivity detectors (C^4^Ds) are more advantageous since they can record any species able to migrate inside an electric field; thus, AA derivatization is not required. The ME-C^4^D sensitivity can be enhanced by optimizing the dimension of the detection electrodes. Xu et al. reported the LOD of 1 × 10^−6^ M for L-lysine, which was achieved by optimizing the electrode length to 550 µm [32]. The ME-C^4^D analysis of L-arginine and L-lysine has been reported by several more authors in studies that were more focused on achieving enhanced separation than on precise quantification. Abad-Villar et al. added Tween 20 in 2.3 M acetic acid BGE to prevent the interaction between analytes and the PDMS microchannel surface [33]. To prevent band broadening, Sydes et al. used the prototype for in-channel potential measurements during injection [34]. Hybrid silicon-on-isolator (SOI) PDMS microchips were proposed to avoid low reproducibility, adsorption of hydrophobic analytes, and weak heat dissipation [32,35].

The aim of this study was to develop and optimize the analytical performance of low-cost and portable ME-C^4^D instrumentation for label-free, simple, and effective analysis of L-arginine, L-ornithine, and L-lysine in nutritional supplements. We proved that biomolecules such as AAs can be analyzed on polymeric material microchips, which are cheaper alternatives to glass microchips and are also more user-friendly. The goal of the study was to optimize the working conditions, enhance analytical performances of the ME-C^4^D analysis, and minimize the negative influence of plastic microchips on the separation resolution, which was demonstrated by the successful separation of L-arginine and L-ornithine. The analytical features were validated and compared with CE–ultraviolet–visible spectroscopy (UV-VIS) and HPLC–diode array detector (DAD) methods. The obtained results showed that ME-C^4^D could be used as a simple tool for the simultaneous analysis of diverse chemical species. The benefits of the developed method were evidenced by calculating its analytical greenness, which was improved compared to CE-UV-VIS and HPLC-DAD analyses.

## 2. Results and Discussion

### 2.1. Optimization of ME-C^4^D Analysis

Reaching optimal electrophoretic conditions is essential in ME analysis since the separation occurs within an exceedingly short separation path, and therefore, it is more difficult to separate analytes efficiently. In addition, the strength of the electrophoresis driving force that can be applied in the form of separation voltage is limited for a short microchannel and depends on the platform, which is still not commercially widely available. Electrophoretic parameters that can be applied during ME, such as the strength of separation voltage, depend on the ionic strength of the BGE. For these reasons, the first step was BGE optimization. For normal polarity electrophoresis of selected AAs, the BGE with approximately pH = 2 is preferable to ensure the Aas are in protonated form. In this study, optimization was performed for the L-arginine standard solution, and the selected conditions were later confirmed as valid for other tested AAs. The examined BGE components were non-toxic, easily available, and inexpensive chemicals. Firstly, single weak organic acids were examined, including 5.01 × 10^−3^ M citric acid (2.3 pH), 9.5 × 10^−3^ M iminodiacetic acid (2.52 pH), 2.4 × 10^−9^ M tartaric acid (5.8 pH), 0.5 M acetic acid, and 9.11 × 10^−3^ lactic acid (2.92 pH). The suitability of BGE composition was fairly difficult to predict since its electrophoretic mobility depends on several factors. Cheng and Yuan proposed a simple model for the prediction of absolute electrophoretic mobility of organic acids, employing only three molecule numerical descriptors: number of acid groups, number of atoms, and molecular weight [36]. The reported absolute mobility increased for lactic acid, acetic acid, tartaric acid, and citric acid, in the order of mention. This was in agreement with the current recorded during the separation step in our study, where a sequential increase was exhibited for BGEs composed of lactic acid, tartaric acid, acetic acid, and citric acid, with corresponding values of 6 μA, 11 μA, 15 μA, and 18 μA, respectively. It can be observed that the BGE consisting of tartaric acid produced a lower current than BGE consisting of acetic acid, while theoretically calculated mobilities according to the proposed approach were reversed. However, in our study, tartaric acid was the only BGE prepared at pH = 5.8. The BGE solutions that consisted of other organic acids were prepared at a lower pH (approximately pH = 2); thus, they were more concentrated, and the produced current was higher as well.

**Figure 1:**
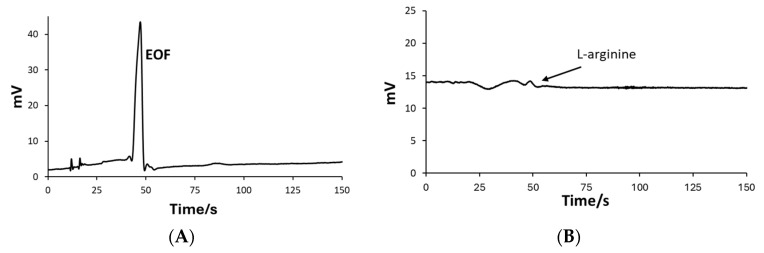

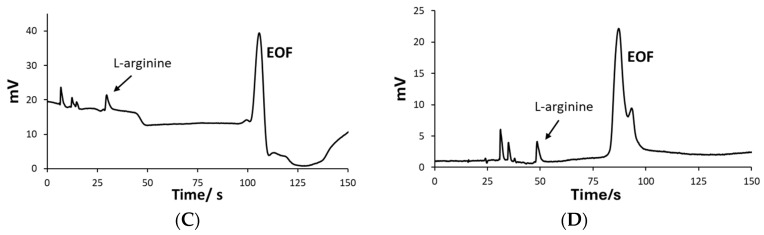
ME-C^4^D electropherograms for analyses of 5 × 10^−5^ M L-arginine standard solution in BGE consisting of (**A**) 2.4 × 10^−9^ M tartaric acid (5.8 pH), (**B**) 5.01 × 10^−3^ M citric acid (2.3 pH), (**C**) 9.11 × 10^−3^ M lactic acid (2.92 pH), (**D**) 9.5 × 10^−3^ M iminodiacetic acid (2.52 pH).

When the lactic acid, tartaric acid, and iminodiacetic acid BGEs were used, the electroosmotic flow (EOF) phenomenon was observed in the form of an unusually large peak (Figure 1). Tartaric acid exhibited high mobility; BGE flow was too high. In that case, the EOF can be very intense, causing accumulation of the BGE in certain areas of the microchannel. This led to abrupt changes in the signals, which were manifested as a large peak on the electropherogram. On the other hand, lactic acid and iminodiacetic acid BGEs had lower mobilities, still, EOF was observed in the electropherogram. Due to the low mobility of BGE, analytes had longer interactions with the microchannel walls, which could cause their adsorption to the microchannel wall. Adsorption of the analyte can alter the local profile of BGE flow and change the C^4^D response. A local change in the BGE flow can also cause a change in the C^4^D response, forming unwanted peaks in electropherograms.

Operating conditions were assessed for all examined BGEs. The frequencies were tested in a range of 100–1200 Hz and amplitude in the 10–80% range. BGEs consisting of citric acid and tartaric acid (Figure 1A,B) gave an unstable baseline and were not usable at any frequency and amplitude. In most cases, peaks were not detectable due to a wriggly baseline. The baselines obtained using lactic acid or iminodiacetic acid were wriggly and unusable as well (Figure 1C,D), although the shape of electropherograms was stable between runs. When lactic acid was used, the AA peaks were not detectable, while BGE, which consisted of iminodiacetic acid, resulted in visible AA peaks, although not appropriate for quantitative measurement.

**Figure 2:**
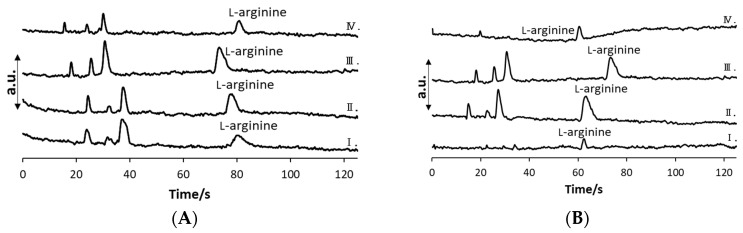
The ME-C^4^D electropherograms of 5 × 10^−5^ M L-arginine standard solutions in (**A**) BGE consisted of 0.2 M acetic acid and different concentration of iminodiacetic acid: (I) 1 × 10^−4^ M, (II) 5 × 10^−5^ M, (III) 1 × 10^−5^ M, and (IV) 5 × 10^−6^ M; and (**B**) BGE consisted of 1 × 10^−5^ M iminodiacetic acid and different concentration of acetic acid: (I) 0.4 M, (II) 0.3 M, (III) 0.2 M, (IV) 0.1 M.

Duong et al. previously tested BGEs consisting of lactic acid, citric acid, and acetic acid for the determination of AAs in royal jelly supplements on reverse polarity CE-C^4^D [19]. Lactic acid and acetic acid were sufficient as BGE components, but their concentrations were considerably higher than those used in this study. For example, baseline fluctuation and baseline drift occurred when lactic acid was prepared in concentrations lower than 2 M. In our investigation, flat baselines and peaks appropriate for the quantitative determination of AAs were obtained only for BGE consisting of acetic acid (Figure 2A). Thus, acetic acid was further considered as a BGE component for electrophoretic measurement. Among the assessed organic acids, acetic acid had the lowest acidity with a 1 to 2 order of magnitude lower *K_a_* value than other organic acids tested in this study. It was still unrevealed if this was an affecting factor that dictated BGE suitability. The BGEs consisting of acetic acid are well-studied for the CE-C^4^D analysis of AAs and are often used due to a stable baseline with minimal drifts [37]. In the current study, the highest conductivity response for L-arginine in BGE consisting of 0.5 M acetic acid was obtained for the 400 Hz applied frequency; however, an overshoot was present. By increasing the detection frequency to 900 Hz, the overshoot was diminished while the peak height decreased insignificantly. The best results were obtained using a 900 Hz operation frequency with 15% amplitude. Since the C^4^D signal of a particular analyte is strongly related to the used detection frequency, the optimization of the operating conditions ensured the method selectivity toward analyzed AAs.

To avoid peak dispersion during ME and CE separations, the mobility of BGE should be as similar as possible to the mobility of the analyte. However, to achieve the highest C^4^D response, the conductivities have to differ enough. Thus, the optimization of BGE used for ME-C^4^D and CE-C^4^D analysis is often challenging. This is expected since both mobility and conductivity are influenced by the same parameter, i.e., by the amount of charged particles. Our previous study on the ME-C4D analysis of AAs showed that the addition of a small amount of a second organic acid to the BGE solution could improve separation, peak shape, and conductivity signal [38]. For this reason, in this study, the BGE consisting of acetic acid as a primary component was further modified as well. Between the tested organic acids, only iminodiacetic acid assured both a stable baseline and visible L-arginine peak and thus was selected for further BGE modification. The influence of acetic acid and iminodiacetic acid concentrations was estimated in the ranges of 0.1–0.5 M and 5 × 10^−6^–1 × 10^−4^ M, respectively, as presented in Table 1.

**Figure 3:**
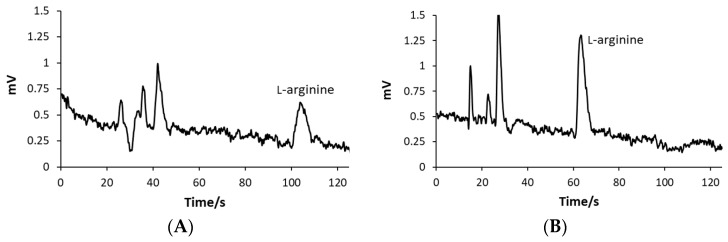
ME-C^4^D electropherogram for analysis of 5 × 10^−5^ M L-arginine standard solution in BGE consisting of (**A**) 0.5 M acetic acid (unmodified BGE) and (**B**) 0.3 M with 1 × 10^−5^ M iminodiacetic acid.

Using all examined BGEs, additional peaks were consistently observed alongside the L-arginine peak (Figure 1 and Figure 2). These additional peaks were identified as system peaks, a characteristic phenomenon in capillary electrophoresis. System peaks arise from localized fluctuations in the concentration of the background electrolyte induced by the migration of the analyte. The highest detector response was achieved for the BGE composed of 0.1 M acetic acid with 1 × 10^−5^ M iminodiacetic acid (BGE 6); however, in BGE 6, the L-arginine peak was irregularly shaped, unreproducible, and EOF was often present. The next highest responses were obtained for 0.2 M acetic acid with 1 × 10^−5^ M iminodiacetic acid BGE (BGE 4), in which the L-arginine peak was well shaped, and thus BGE 4 was firstly selected for analysis. Although BGE 4 offered the best analytical performance, the EOF was frequently present during analysis because of the low ionic strength. Hence, BGE consisting of 0.3 M acetic acid with 1 × 10^−5^ M iminodiacetic acid was selected since it enabled reproducible analysis. It can be noticed that the modification of the single-component BGE (BGE 1) had a strong impact on improving detector response, reducing migration time, and raising the migration time repeatability (Table 1). Modifying the concentration of acetic acid and by adding 1 × 10^−5^ M iminodiacetic acid as a secondary organic acid, the detector response for L-arginine increased by 114.89%, and the migration time was reduced to 63.4 s (shown in Figure 3). It is assumed that the lower ionic strength of the modified BGE contributed to the higher zeta potential and, therefore, to higher electroosmotic flow. The generated current in the modified BGE was 20 μA, which was about 2 μA higher than the unmodified 0.5 M acetic acid. As selectivity in ME-C^4^D analysis is strictly dependent on migration time repeatability, the BGE modification notably contributed to the more selective analysis as well. The standard deviation (SD) of L-arginine migration time in the optimized buffer (BGE 7) was 0.30 s, while in the unmodified buffer (BGE 1), SD was 2.96 s.

The influence of electrophoretic conditions was assessed using optimized BGE and the detection parameters. The detection sensitivity can be enhanced by applying the most proper injection settings; thus, injection time and voltage were examined first. The influence of injection time was investigated from 5 to 25 s, using a 5 s step increment. The peak height and peak area of L-arginine increased by elevated injection time up to 20 s. The peak broadening was noted for injection time greater than 20 s. Due to hydrodynamic and diffusion effects, sample leakage often occurs from the microchannel’s intersection toward the separation microchannel. Utilizing prolonged injection times, a greater amount of sample diffuses, leading to a broadened sample zone. The influence of injection voltage was studied over a range of +1500 to +2750 V, with increments of +250 V. The selected injection parameters were +1000 V, and injection voltage was applied for 20 s. The voltage variations for the separation step were examined within the range of +1500 to +2750 V, with increments of +250 V. As expected, the higher separation voltage contributed to lower migration time, higher peak symmetry, and pronounced peak height. For this reason, the +2750 V was selected for separation voltage. The optimization experiments of the separation step were conducted at separation times of 120, 180, and 240 s. The 120 s separation time was selected as sufficient for all tested analytes to reach the detector and also ensured no appearance of EOF.

### 2.2. ME-C^4^D Method Validation

The performance of the proposed ME-C^4^D method was validated via the assessment of different analytical characteristics (summarized in Table 2) involving repeatability, sensitivity, selectivity, and linearity of C^4^D response. To determine the dependence of C^4^D response to the concentration of analytes in ME-C^4^D analysis, the values of the peak height or peak area can be used. Usually, the peak area is preferable; however, the lower RSD values were obtained for peak heights, and thus, peak heights were used for linear regression analysis. The RSD values of peak heights, peak areas, and migration times for all analyzed analytes are shown in Table 2.

As shown in Table 2, the linear responses of the method were high within a concentration range of one order of magnitude. The experimental detection limits (LOD_e_) were 7.5 × 10^−6^ M, 7.5 × 10^−6^ M, and 5 × 10^−6^ M for L-arginine, L-ornithine, and L-lysine, respectively. Most studies on ME analysis of AAs do not report the LOD and LOQ values and only demonstrate the ME separation of AA model solutions of certain concentrations. For instance, ME-LIF analyses of 2 × 10^−3^ M and 2.5 × 10^−5^ M L-arginine model solutions have been reported without estimated LOD and LOQ values [39,40]. Also, the LOD and LOQ values cannot be found in two other papers, where ME-amperometry analyses of 2.64 × 10^−3^ M and 1.6 × 10^−3^ M L-arginine model solutions were performed [28,41].

So far, the highest sensitivities in ME analysis have been reached by LIF detection [25]. The reported LODs for L-arginine were between 3.6 × 10^−9^ M and 3.29 × 10^−5^ M [42,43,44,45]. Among the AAs investigated in this study, the lowest LOD value found in the literature was 2.5 × 10^−10^ M for L-lysine. This was achieved by applying additional steps of field-amplified stacking and reversed-field stacking that enabled analysis with an enrichment factor of 165 [25]. Different sensitivities have been achieved for ME-LED-induced fluorescence analysis of L-arginine; the reported LODs were 5 × 10^−8^ M and 1 × 10^−3^ M [46,47]. The reported LODs for ME-amperometry analysis of L-arginine were between 3.2 × 10^−6^ M and 1.36 × 10^−5^ M, which were close to those obtained in this study [27,29,31]. The LOD values for ME-LED-induced fluorescence and ME-amperometry analyses of L-lysine and L-ornithine were not found.

There are only a few papers describing the ME-C^4^D analysis of L-arginine, L-ornithine, and L-lysine. The literature overview of the ME-C^4^D methods applied for the analysis of different AAs can be found in Table 3. From Table 3, it can be observed that the detection limits reported by other authors were similar to those obtained by the presented study. The achieved detection limits indicated the possible application of the ME-C^4^D instrumentation in the analysis of real samples of nutritional supplements. Thanks to the short microchannel, optimized BGE, and other electrophoretic conditions, the migration times of AAs were less than 2 min, which enabled rapid monitoring of the sample. The shortest migration time of 52.4 ± 0.6 s was observed for L-ornithine, which indicates that it had the highest electrophoretic mobility among the tested AAs. At the pH = 2.59 of the used BGE 7, the dominant form of all analytes was the +2 cationic form; thus, the lowest molar mass of L-ornithine enabled its highest mobility. The RSD values of migration times that were presented in Table 2 showed the absence of fluctuations in the migration time of AAs between runs, thus confirming method reproducibility and also method selectivity. C^4^D detector records any conductive species; thus, migration time repeatabilities were important for selective analysis.

When nutritional supplements are analyzed, the presence of other ions, including active ingredients (such as metal cations and vitamins), excipients, and contaminants, may influence the selectivity of the method. For that reason, the real samples of nutritional supplements were spiked with a standard solution of AAs, and an increase in the C^4^D signal confirmed the method’s selectivity.

The migration times of L-lysine and L-arginine were similar, and thus, the selectivity of the method was not possible to test in samples that contained both of these AAs. Nevertheless, that demonstration was not crucial as nutritional supplements containing only L-lysine and L-arginine cannot be found on the market. Thus, selectivity was demonstrated by the simultaneous analysis of L-arginine and L-ornithine, the mixture that is frequently sold. Although L-arginine and L-ornithine have almost identical chemical properties, their complete separation was possible on ME-C^4^D, and therefore they could be identified.

**Figure 4:**
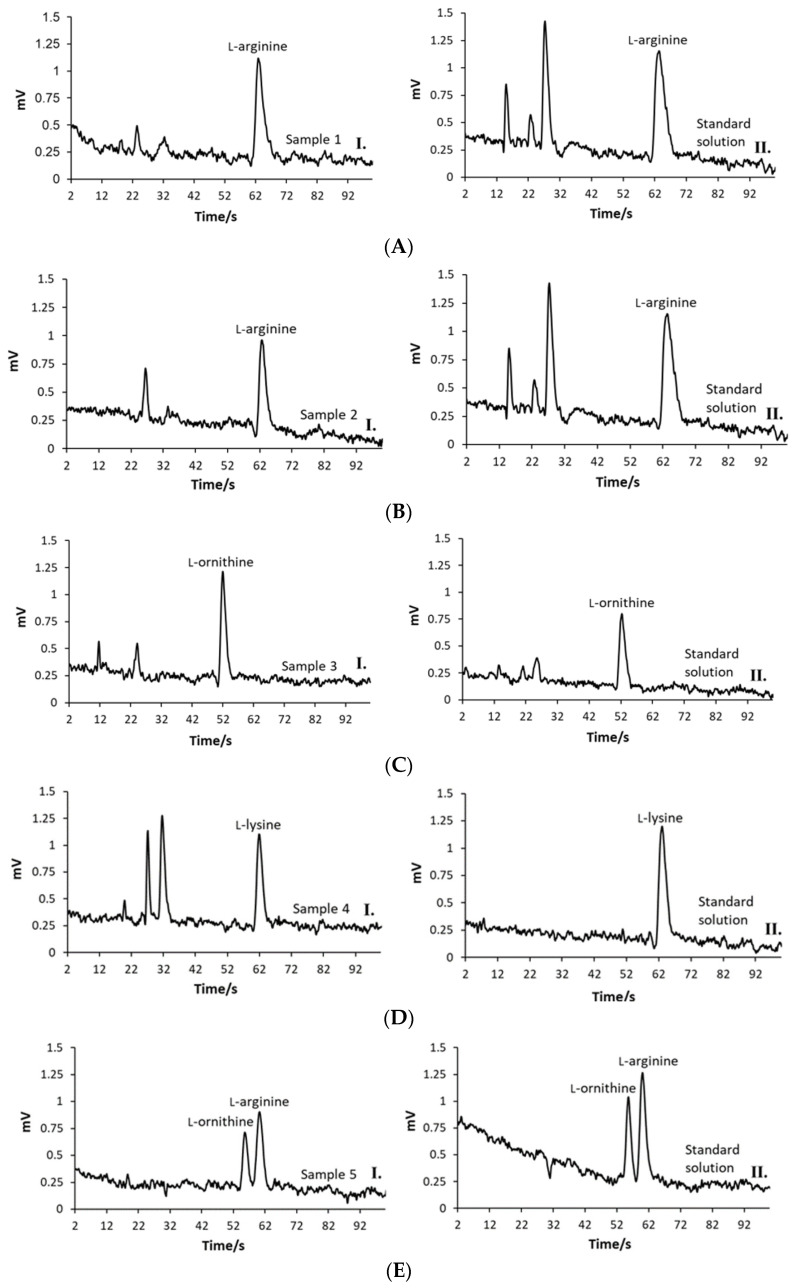
ME-C^4^D electropherogram for analysis of (**A**)(I) Sample 1, (**A**)(II) 5 × 10^−5^ M L-arginine standard solution, (**B**)(I) Sample 2, (**B**)(II) 5 × 10^−5^ M L-arginine standard solution, (**C**)(I) Sample 3, (**C**)(II) 2.5 × 10^−5^ M L-ornithine standard solution, (**D**)(I) Sample 4, (**D**)(II) 5 × 10^−5^ M L-lysine standard solution, (**E**)(I) Sample 5, (**E**)(II) the mixture of 5 × 10^−5^ M and L-arginine and 2.5 × 10^−5^ M L-ornithine standard solution.

According to the migration times visible in the electropherogram in Figure 4E(II), the identification of AAs was confirmed by spiking the sample with individual AAs and monitoring the detection signal increase. The method was able to analyze each AA accurately within a complex mixture without mutual interference, which was confirmed by recovery experiments.

### 2.3. Application of the New ME-C^4^D Method for the Quantification of L-arginine, L-ornithine, and L-lysine in Nutritional Supplements

The performance of the analytical methodology developed during this investigation was further confirmed by testing its applicability in analyzing real samples of nutritional supplements. The AA content was evaluated in five different nutritional supplements bought in local pharmacies and web shops. All samples were prepared as described in Section 3.2 and then analyzed with ME-C^4^D devices under conditions selected during the optimization process. The sample preparation protocol did not include any pre-concentration steps or derivatization processes, while the purifying step included only simple filtration of the sample solutions using cellulose syringe filters. Since nutritional supplement samples contained a variety of sub-ingredients (see Section 3.1), the AA peaks were authenticated by spiking the samples with AA standard solutions. The run times established during the analysis of AA standard solutions sometimes need to be extended during the analysis of the samples with the complex matrices to avoid the occurrence of EOF that can disturb the analysis. However, in the present trial, a sample matrix did not cause the EOF. The selected run time of 120 s was long enough for the analysis of all real samples. The recorded sample’s electropherograms are presented in Figure 4.

The AA signals were easy to read; moreover, they were symmetric, sharp, and without tailing. The migration times of AAs could be affected by the sample matrix, and thus, the method selectivity could be reduced. However, the migration times of AAs in all analyzed samples were consistent with data already recorded for standard solutions. The L-arginine migration times of 63.4 ± 0.4 s and 62.8 ± 0.3 s in analyzed Sample 1 and Sample 2 were the same as already recorded for the standard solution where the migration time was 63.4 ± 0.4 s. Also, the electropherogram for Sample 3 showed an L-ornithine peak at 51.9 ± 0.9 s, while the already evidenced value was 52.4 ± 0.6 s. Sample 4 contained L-lysine as the active ingredient, which reached the detector at 63.0 ± 1.0 s, while the previously recorded value was 63.6 ± 0.9 s. The migration times of the L-arginine and L-ornithine in Sample 5 were 61.4 ± 1.3 s and 53.6 ± 1.3 s, respectively. The behavior where L-arginine migrates faster and L-ornithine slower in a mixture compared to single AA samples can be explained by changed interactions between analytes and BGE but also by interactions formed between L-arginine and L-ornithine. For example, due to the guanidinium group, L-arginine can interact with acetate ions more strongly than L-ornithine, and as a result, more ions are concentrated around L-arginine, leading to slower mobility of L-ornithine. On the other hand, hydrogen bonds and electrostatic and ionic interactions formed between the L-arginine and L-ornithine contributed to their more coordinated migration. The AAs in Sample 5 were adequately separated to ensure quantification, visible in Figure 4E(I). The resolution for the separation of AAs in Sample 5, calculated according to Equation (1), was *R* = 1.41. The separation efficiencies calculated according to Equation (2) were *N* = 2688.42 and *N* = 2833.28 for L-arginine and L-ornithine, respectively.

*R* = 2 × (t_2_ − t_1_)/(w_2_ + w_1_) (1)

*N* = 5.54 × (t (s)/w (min)^2^) (2)

t = migration time (min);
w = the width of peak base (min).

For dosages in pharmaceutic forms, variance from the declared value up to 10% is considered acceptable [52]. Still, legislation regarding the tolerance range of deviations in labeling the AA content in nutritional supplements has not been introduced. The results of the nutritional supplements analysis using the herein-developed method showed insignificant disagreement with declared values, which ranged from 2.55 to 7.52%. Those results were further supported by additional sample analysis by CE-UV-VIS and HPLC-DAD instrumentations. The results of AA measurements by all used analytical methods are summarized in Table 4. Statistical comparisons based on *t*-tests at a 95% confidence level did not show a significant difference between the results obtained with ME-C^4^D and CE-UV-VIS nor HPLC-DAD analysis. In all cases, the calculated t-values (t_calc_) were under the critical t-values (t_ctrit_), manifesting the adequate accuracy of the new microfluidic method. All data related to the *t*-test are presented in Table 5. From t_calc_ values shown in Table 5, it can be noticed that in most cases, a higher correlation was found between measured concentrations by ME-C^4^D and CE-UV-VIS compared to results between concentrations measured by ME-C^4^D and HPLC-DAD. The only exception was Sample 4, where the t_calc_ was higher in the case of HPLC-DAD analysis. The results between CE-UV-VIS and HPLC-DAD were also compared, and statistically significant differences were found only for the measured value of L-ornithine in Sample 3.

The accuracy of the method was also investigated through recovery experiments measuring concentrations of AAs that were 20% and 40% higher than values recorded for unspiked samples. The recovery values ranged from 92.75 ± 1.35% to 108.45 ± 1.65%. More information on the results of recovery experiments is presented in Table 6.

### 2.4. The Overall Greenness of All Used Methods Calculated with AGREE Software

To understand the potential benefits of the developed ME-C^4^D method, the comparison with other analytical techniques that were used during this study was discussed, with special emphasis on meeting green analytical chemistry (GAC) principles. The greenness of the methods was calculated using the AGREE software (Version 0.5) and results were presented in the form of a pictogram whose middle depicts the numerical overall score. The score ranges inside a 0–1 scale, where the value is 0, and the red color represents the minimum greenness of the method. Opposite to that, a value of 1 and the dark green color represent the maximum greenness of the method. The marginal part of the pictogram contains 12 sections denoting 12 estimated criteria of GAC principles. The color of each section indicates the performance of each presented GAC principle for the evaluating analytical method.

**Figure 5:**
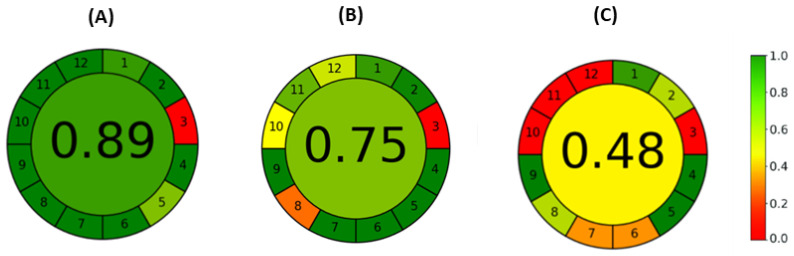
Results of overall greenness by AGREE analysis for (**A**) ME-C^4^D analysis, (**B**) CE-UV-VIS analysis of AAs, and (**C**) HPLC-DAD analysis of AAs. The third input criteria are set as an offline analytical method. The 1–12 refers to greenness score for each of 12 green analytical chemistry principles [54].

The overall greenness obtained for all used methods is shown in Figure 5. The obtained results showed that the ME-C^4^D method was 28.09% greener than the CE-UV-VIS method and 58.43% greener than the HPLC-DAD method. A considerably higher score of greenness was achieved for the ME-C^4^D analysis (0.87) than for the HPLC-DAD analysis (0.48). The HPLC-DAD consumed a large volume of organic solvents and, therefore, generated unwanted waste, some of which, such as acetonitrile and methanol, are toxic. Besides, HPLC-DAD analysis utilized a high-priced separation column, which, in addition to the employed instrumentation and chemicals, highly raised the overall analysis costs. The retention times of L-arginine, L-ornithine, and L-lysine were 355.2 ± 7.8 s, 334.8 ± 12.6 s, and 408.0 ± 6.6 s, respectively, which were more than double longer in comparison to migration times of AAs on microchips. Although the sample derivatization step in HPLC-DAD analysis was online and automatized, it further lowered the analysis throughput. For these reasons, ME-C^4^D is more favorable for the analysis of AAs. In the study conducted by Duriš et al., the AGREE software (Version 0.5) was applied to estimate the greenness of the method developed for the analysis of different pharmaceutical ingredients [53]. The same as in our study, the highest greenness score of 0.81 was obtained for the method employing microchips (microchip isotachophoresis) conjugated to the conductivity detector, while HPLC methods resulted in greenness scores of 0.62 and 0.59 for DAD and MS detection, respectively.

Both electrophoretic methods used during this study showed a high degree of greenness, noticeable in Figure 5. However, the greenness score was still higher for the ME-C^4^D analysis (0.89) than for the CE-UV-VIS analysis (0.75). In both methods, the samples were prepared by simple dilution with water; the purification process included only simple filtration, and the derivatization step was not needed. The number of chemicals used was minimized; indeed, only microliters of samples and BGE solutions were required. From the pictogram, it can be observed that the only advantage of the CE-UV-VIS over the ME-C^4^D is automated instrumentation (5. the GAC principle). However, the ME-C^4^D method is miniaturized (5. the GAC principle) and, therefore, less expensive, while the consumption of energy, samples, reagents, and solvents is minimized. Also, short microchips are easy to operate and take less space than capillaries. Because of lower analysis throughput (8. GAC principle), the overall greenness of the CE-UV-VIS method is lowered. Our study demonstrated that analysis time can be reduced from 15 min to 2 min if the ME-C^4^D is used instead of the CE-UV-VIS. The migration times of AAs in CE were between 678.2 s and 754.9 s, which can be observed in Table 2 and the CE-UV-VIS electropherograms presented in the Electronic Supplementary in Figure S1. Additionally, using C^4^D instead of spectrometric detectors, AAs can be detected simultaneously with other chemical species in one run and without using labels. It should also be mentioned that in the ME-C^4^D analyses, toxic chemicals were not used, and all chemicals were obtained from renewable sources. On the other hand, in the CE-UV-VIS analysis, only copper sulfate pentahydrate was not a green chemical since it is corrosive and toxic to aquatic wildlife and was not obtained from renewable sources. As a result, the 10., 11., and 12. GAC principles were only partially met by the CE-UV-VIS analysis and fully by the ME-C^4^D analysis.

**Figure 6:**
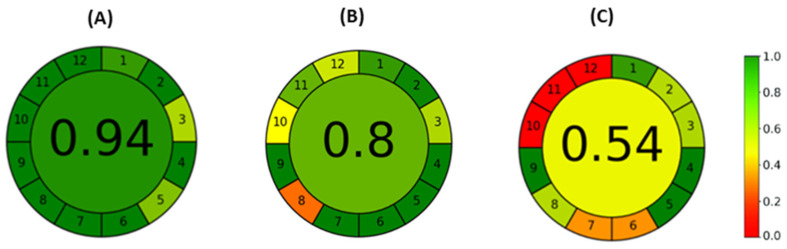
Results of AGREE analysis for (**A**) ME-C^4^D analysis, (**B**) CE-UV-VIS analysis of AAs, and (**C**) HPLC-DAD analysis of AAs. The third input criteria are set as an online analytical method (hypothetical estimate). The 1–12 refers to greenness score for each of 12 green analytical chemistry principles [54].

Using the AGREE program to calculate the greenness of ME-C^4^D analysis, the only lower output (red color) was obtained for GAC principle 3, but this lower output was also obtained for the other analytical methodologies used in this study. However, the red color was expected since GAC principle 3. implies that in situ measurements should be performed. In contrast, all measurements in this study were performed offline. This can not be considered a notable drawback of the method since the ME-C^4^D instrumentation is still used only in research laboratories for the investigation of its potential applications. However, it could be beneficial to upgrade the ME-C^4^D instrumentation further to integrate in situ measurements directly into the manufacturing process as an in-line method of analysis. To assess how significant upgrading the ME-C^4^D instrumentation for integration in the manufacturing process is, the overall method greenness was further calculated using the “online method” as an input parameter for GAC principle 3. in AGREE software. In the same way, the greenness of the online CE-UV-VIS and HPLC-DAD methods was also calculated. The greenness score for hypothetically integrated methods in the manufacturing process is shown in Figure 6. As in the above-described greenness assessment for offline analyses, the highest score was also obtained for the ME-C^4^D (0.94), followed by the CE-UV-VIS (0.8), and finally, with the HPLC-DAD (0.54) method.

The overall greenness for the ME-C^4^D, CE-UV-VIS, and HPLC-DAD methods was enhanced by 5.6%, 6.7%, and 12.5% in comparison to previous results for online methods. From all described, it could be stated that ME-C^4^D was a more convenient method for the AA analysis concerning GAC principles than CE-UV-VIS and HPLC-DAD. Further upgrading of the ME-C^4^D instrumentation, for example, the introduction of automation could be favorable in the nutritional supplement industry, not only for AA analysis but also for vitamins, minerals, and impurity analysis.

## 3. Materials and Methods

### 3.1. Chemicals and Materials

All chemicals used in this study were analytical reagent grade. L-arginine, L-ornithine-HCl, L-lysine-HCl, acetic acid, citric acid, glycolic acid, iminodiacetic acid, and tartaric acid, all from Sigma-Aldrich (St. Louis, MO, USA). Copper (II) sulfate was obtained from T.T.T. Ltd. (Novaki, Croatia). Five different nutritional supplements with the declared content of L-arginine, L-ornithine-HCl, and L-lysine were purchased in local pharmacies and web shops; Sample 1 was tablet dosage, Sample 2 was powder dosage, and Samples 3, 4, and 5 were capsule dosage. In addition to the AAs, the sample contained some other active ingredients such as minerals (Ca, Zn, Cu) and vitamins (B_6_, B_9_, B_12_, K, C) and supporting ingredients (microcrystalline cellulose, hypromellose, stearic acid, silicon dioxide, cellulose gum, magnesium stearate, methylcellulose, dicalcium phosphate, glycerin, citric acid, malic acid, maltodextrin magnesium carbonate, sodium bicarbonate, raspberry flavor, lime flavor, sucralose, acesulfame potassium, carmine, medium chain triglycerides, silicon dioxide, magnesium oils of fatty acids), or organic extract (rice extract mixture, Chinese cinnamon). Exact data of the concentration of the target analytes for each supplement are noted in the result section in Table 4.

### 3.2. Sample Preparation

The procedure for the sample preparation was the same for all nutritional supplements, apart from final dilution. The contents of the capsules were poured out before weighing, while the tablets were crushed. For cross-section sampling, ten sample portions were homogenized in a mortar, and then a mass of 10 mg was dissolved in 10 mL of ultrapure water. Thus, prepared solutions were filtered with cellulose syringe filters (0.22 μm pore diameter, (Marrickville, NSW, Australia, Labex Ltd.)) and then diluted to obtain the concentration of AAs within the linear concentration range. Before analyzing the sample, solutions were sonicated to remove gases.

### 3.3. ME-C^4^D Analysis

A compactly integrated kit with an ET121 (eDAQ) device, a High Voltage Sequencer (HVS), and an ER225 C^4^D was used for ME-C^4^D analysis. The C^4^D detection electrodes were located on the ET121 platform, which served as a bench for the ME separation microchip and was cable-connected to the ER225 C^4^D system. The C^4^D signal was monitored using PowerChrom (eDAQ, Version 2.7.8, Denistone East, NSW, Australia). For sample injection and separation, the HVS unit was used as a power supply, which was controlled by/via Sequencer software (EDAQ, Version 1.3.3, Denistone East, NSW, Australia). The HVS has four ports initiated for connecting high-voltage electrodes, which were placed in microchip reservoirs during analysis. The utilized microchip was a double-T poly(methyl methacrylate) microchip (Chip-Shop GmbH, Jena, Germany) with a separation microchannel of 7.2 cm effective length and 50 × 50 µm cross-section.

#### 3.3.1. The Microchip Preparation Procedure

Before operating the microchip device, the microchip was placed on the ET121 platform so that C^4^D electrodes were near the end of the separation microchannel, touching only the exterior microchip side; thus, microelectrodes were detached from the microchannel and BGE solution. Then, microchannels were rinsed with ultrapure water, followed by rinsing with BGE solution. Before analysis, microchannels and reservoirs were filled with fresh BGE solution, and the system was tested by recording a blank run. For analysis, 50 μL of the sample solution was loaded in the sample reservoir (R1), and 50 μL of BGE solution was loaded in each of the remaining three reservoirs (sample waste reservoir (R3), BGE reservoir (R2), and BGE waste reservoir (R4)). When the assay was ready, the high-voltage electrodes were placed in each microchip reservoir. The AA quantification was accomplished based on three measurement series, each of which included five repetitive runs. A microchip was rinsed with BGE solution between each series and with ultrapure water at the end of measurements.

#### 3.3.2. The ME Injection and Separation Procedure

**Figure 7:**
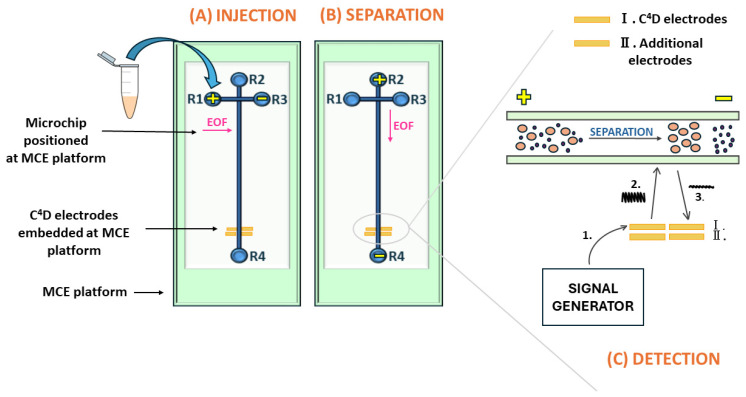
Visual representation of (**A**) injection, (**B**) separation, and (**C**) detection step in ME-C^4^D analysis. The pink arrows represent the direction of electroosmotic flow (EOF). Different colors illustrate various parts of the ME instrumentation: green denotes the ME platform, gray represents the microchip, blue highlights the injection and separation microchannels and reservoirs, and yellow indicates the C^4^D electrodes. The plus and minus signs denote the applied voltage.

Microchip floating injection was performed by applying a +1000 V for 20 s across the injection microchannel located between the sample reservoir (R1) and sample waste reservoir (R3) (Figure 7). Next, the sample was continuously driven from the sample reservoir (R1) to the sample waste reservoir (R3), whereas the produced current during this step was recorded as 40 μA. The injection step required control of only two of the overall four microchip reservoirs. The termination of this step implies the end of the voltage application between the sample reservoir (R1) and the sample waste reservoir (R3). Then, voltage was applied between the BGE reservoir (R2) and BGE waste reservoir (R4) to perform the separation. Thus, the generated electric field pushed the sample from the T-intersection toward the detection part of the separation microchannel. In this study, the separation was performed at +2750 V applied to the BGE reservoir (R2) and ground to waste reservoir (R4) for 260 s.

#### 3.3.3. The C^4^D Detection of AAs

In the C^4^D detection system, two pairs of electrodes were used, the first one (Figure 7(I)) for detection and the second one (Figure 7(II)) to nullify the stray capacitance generated by pairing detection electrodes. At the point when AAs reach the detection electrode, they are detected at 900 kHz and 15% amplitude. The capacitive signal was shifted from the transmitter electrode to liquid inside the microchannel and then to the receiving electrode. After the transiting signal through the solution, it was decreased depending on the solution’s conductivity. In the presented experiments, the used BGE had higher conductivity than AAs, and therefore, negative signals were inversed to obtain positive AAs peaks.

### 3.4. CE-UV-VIS Analysis

CE-UV-VIS analysis was accomplished on Agilent Technologies 7100 CE System coupled to UV-DAD (diode-array detector) (Waldbronn, Germany). The data acquisition was performed with 3D-CE ChemStation software (B. 04.00. and Version 7.01, Agilent Technologies). The AAs were injected using 50 mbar injection pressure for 5 s and then separated inside an uncoated fused-silica capillary (41.2 cm effective length and 50 μm ID, sustained at 25 °C) by applying +15.00 kV separation voltage. The analysis protocol was operated as described by Jiang et al. [55] and was selected since used BGE consisted of 5 × 10^−5^ M CuSO_4_ and 0.05% acetic acid (pH = 4.5), from which Cu (II) ions interacted with AAs via coordination chemistry. Those interactions are detectable at 254 nm, and therefore, AAs can be detected without using the tedious process of sample labeling. At the beginning of measurements, it was electrokinetically conditioned as follows: ultrapure water (5 min), NaOH (5 min), ultrapure water (5 min), and BGE (5 min). Between each run, the capillary was conditioned with BGE (5 min), while at the end of the working day, with ultrapure water (5 min), 0.1 M NaOH (5 min), and again ultrapure water (10 min).

### 3.5. HPLC-DAD Analysis

The HPLD-DAD analyses were carried out on the Agilent 1260 Infinity II HPLC system (Agilent, Santa Clara, CA, USA) interfaced with DAD, quaternary pump system, autosampler, and column department. Data acquisition was performed using the Agilent OpenLAB CDS software (v. 2.6, Agilent, Santa Clara, CA, USA). The procedure was performed as described in technical tones written by Steed [56]. AAs were derivatized with phthalaldehyde using a 2 min reaction and then separated inside the 4.6 × 100 mm Poroshell 120 EC-C18 column (Agilent, Santa Clara, CA, USA) operated at 40 °C (exit side at 35 °C). Mobile phase A (10 mM Na_2_HPO_4_–10 mM Na_2_B_4_O_7_, pH = 8.2) and mobile phase B (45 ACN: 45 MeOH: 10 H_2_O by volume) were used to elute samples. The gradient elution system was: 1–7 min, 2% MP B; 7–7.1 min 57% MP B, 7.1–8.4 min, 100% MP B; 8.4–8.6 min, 100% MP B; 8.6–8.7, 2% MP B. The DAD acquisition wavelength was set at 338 nm, and samples were recorded through the bandwidth of 10 nm (Ref 390 nm and 20 nm) in 0.00 min and at 262 nm through the bandwidth of 16 nm (Ref 324 and 8 nm) in 5.53 min.

### 3.6. Software for Calculation of Analytical Greenness of the Used Analytical Methods

The “AGREE” software was used to calculate the greenness of all used analytical methods during this study. The software contains 12 input criteria referring to the principles of GAC. The weight of each parameter can be rated from 1 to 4 (selected value), depending on parameter importance in a particular analysis. A more detailed description of each principle and the AGREE software can be found in a paper published by Pena-Pereira et al. [57]. To calculate the greenness of methods, the input criteria were set to the default, giving the same weight for each GAC principle.

## 4. Conclusions

In this study, we developed a new microfluidics method for the analysis of AAs on compactly integrated ME-C^4^D instrumentation. Improved C^4^D response and reduced migration times of AAs were accomplished by detailed optimization of the composition of the BGE and electrophoretic parameters. The best method performance was achieved using BGE composed of 0.3 M acetic acid with 1 × 10^−5^ M iminodiacetic acid, applying 20 s injection time and 2.75 kV separation voltage. Using the proposed conditions, the LOD for L-ornithine was 5 µM, and for L-arginine and L-lysine was 7.5 µM. The minimized sample preparation process and short migration times between 52.4 s and 63.6 s resulted in high-speed analysis. The applicability of the method was demonstrated by the determination of L-arginine, L-ornithine, and L-lysine in different commercial nutritional supplements.

To our knowledge, this is the first study that successfully utilizes ME-C^4^D instrumentation to analyze L-arginine, L-ornithine, and L-lysine in real samples, and the first study in the application of the ME technique for analyzing L-arginine, L-ornithine, and L-lysine in nutritional supplement samples.

The results of the nutritional supplement analysis were compared with results obtained from the CE-UV-VIS and HPLC-DAD analyses, concluding the high accuracy of the method. The degree of greenness for all utilized methods was compared, and the best greenness performance was obtained for the ME-C^4^D method. Hence, ME-C^4^D is favorable for the analysis of AA samples, especially when high analysis throughput is in major demand. The progress in ME-C^4^D instrumentation for integration into the nutritional supplement manufacturing process could contribute to decreased misleading information on AA concentrations shown in nutritional supplement declaration.

## Figures and Tables

**Table 1:** The data on migration time and peak response of L-arginine using unmodified and modified acetic acid BGEs.

	BGE Component (M)		Migration Time (s)	Peak Height (mV)	Peak Area (mV s)
	Acetic Acid	Iminodiacetic Acid			
BGE 1	0.5	-	105.05 ± 2.96	0.47 ± 0.04	3.04 ± 0.45
BGE 2	0.2	1 × 10^−4^	78.49 ± 2.12	0.51 ± 0.07	2.33 ± 0.77
BGE 3	0.2	5 × 10^−5^	78.86 ± 0.50	0.69 ± 0.02	2.21 ± 0.34
BGE 4	0.2	1 × 10^−5^	70.52 ± 2.35	0.87 ± 0.04	3.81 ± 1.24
BGE 5	0.2	5 × 10^−6^	81.08 ± 1.92	0.54 ± 0.06	1.37 ± 0.24
BGE 6	0.1	1 × 10^−5^	61.04 ± 8.77	1.57 ± 0.21	6.37 ± 2.15
BGE 7	0.3	1 × 10^−5^	63.44 ± 0.30	1.01 ± 0.04	3.65 ± 0.14
BGE 8	0.4	1 × 10^−5^	63.38 ± 0.93	0.48 ± 0.03	0.72 ± 0.06

n = 5.

**Table 2:** Analytical characteristics of the ME-C^4^D, CE-UV-VIS, and HPLC-DAD methods for the analysis of L-arginine, L-ornithine, and L-lysine.

	ME-C^4^D	CE-UV-VIS	HPLC-DAD
L-arginine			
Migration/retention time (s)	63.4 ± 0.4	754.9 ± 20.5	355.2 ± 7.8
RSD_area_ (%)	3.86	5.20	3.95
RSD_height_ (%)	3.74	3.93	N/A
RSD_time_ (%)	0.47	2.71	2.20
R^2^	0.9971	0.9902	0.9982
Linear range (µM)	10–100	1150–5740	9–90
Slope	16,732	178.81	7.03
Intercept *	0.162	−0.0678	1.85
LOD_t_ (µM)	0.19	N/A	2.50
LOQ_t_ (µM)	0.64	N/A	8.33
LOD_e_ (µM)	7.5	N/A	N/A
LOQ_e_ (µM)	10.0	N/A	N/A
L-ornithine			
Migration/retention time (s)	52.4 ± 0.6	706.5 ± 4.3	334.8 ± 12.6
RSD_area_ (%)	3.28	7.85	5.21
RSD_height_ (%)	2.88	3.98	N/A
RSD_time_ (%)	0.93	0.60	3.76
R^2^	0.9989	0.9978	0.9967
Linear range (µM)	7.5–75	1190–4740	9–90
Slope	21,753	193.71	2.13
Intercept *	0.1613	11.971	−43.3
LOD_t_ (µM)	0.15	N/A	1.2
LOQ_t_ (µM)	0.50	N/A	4.0
LOD_e_ (µM)	5.0	N/A	N/A
LOQ_e_ (µM)	7.5	N/A	N/A
L-lysine			
* Migration/retention time (s)	63.6 ± 0.9	678.2 ± 0.4	408.0 ± 6.6
RSD_area_ (%)	7.41	4.34	2.71
RSD_height_ (%)	4.56	2.91	N/A
RSD_time_ (%)	1.09	0.05	1.6
R^2^	0.9992	0.9930	0.9962
Linear range (µM)	10–100	1090–4380	9–90
Slope	19,790	171.67	0.47
Intercept	0.1429	1.2473	−0.73
LOD_t_ (µM)	0.18	N/A	2.5
LOQ_t_ (µM)	0.60	N/A	8.25
LOD_e_ (µM)	7.5	N/A	N/A
LOQ_e_ (µM)	10.0	N/A	N/A

* Mean ± standard deviations (n = 3); LOD—defined as S/N = 3; LOQ—defined as S/N = 10.; n = 5 (ME-C^4^D) and n = 3 (CE-UV-VIS) for calculated SD and RSD values. Abbreviations: N/A, not available; LOD_e,_ experimental limit of detection; LOD_t,_ theoretical limit of detection; LOQ_e_, experimental limit of quantification; LOQ_t_, theoretical limit of quantification.

**Table 3:** A literature overview of the ME-C^4^D method for analysis of AAs.

Analyte/Sample	BGE	Chip Material	L_T_/L_E_, (cm)	Analyzed Concentration (µM)	Linear Concentration Range/Tested Concentration Range	LOD (μM)	Reference
Trp, Phe, Thr, Tyr/model solution	10 mM CAPS/50 mM AMP (pH = 10.8)	PMMA	8/7	100	N/A	32–50	[48]
Trp, Glu, Asp/model solution	10 mM CAPS/50 mM AMP (10.8)	PMMA	8/7	5–10	N/A	N/A	[49]
Lys, Arg, Gly, Ala, Val, Ile, Ser, Thr, Met, Gln, Phe, Pro/model solution	2.3 M acetic acid (pH = 2.1) + 0.05% Tween 20	PMMA	8.5/7.5	250–500	N/A	50 (Gln) 12 (Arg)	[33]
3-MH 1-MH His/urine	20 mM MES + 5 mM LiOH + 1% *m*/*v* dextran (pH = 5.5)	silicon	5.3/3.8	118.22 128.91	59.11–591.09 64.45–644.54	18.3–26.4	[24]
Gly, His, Lys Glu/model solution	15 mM MES/15 mM His (pH = 6.1)	glass	-/9	2500	N/A	N/A	[50]
Lys, Arg, His, Gly, Ala, Val Leu, Thr, Met, Trp, Phe, Tyr Cys/model solution	2 M acetic acid (pH = 2.25)	PEEK	9.5/-	400–800	N/A	N/A	[51]
Arg, Lys, Gly/model solution	10 mM MES/10 mM His (pH = 5.4)	glass/PDMS	4/-	100	N/A	10–20	[35]
Lys, Phe/model solution	10 mM borate	SOI-PMMA	3/-	100	N/A	1	[32]

Abbreviations: AMP, Adenosine monophosphate; CAPS, 3-(Cyclohexylamino)-1-propanesulfonic acid, L_T_/L_E_, total length/effective length; 1-MH, 1-methyl-histidine; 3-MH, 3-methyl-histidine; MES, 2-(*N*-morpholino)ethanesulfonic acid, N/A, not available; PMMA, poly(methyl methacrylate); PEEK, polyether ether ketone.

**Table 4:** Results of the quantitative determinations of AAs in nutritional supplement samples.

SAMPLE No. (Analyte)	* Declared Value (mg/g Sample)	Results	ME-C^4^D Analysis	CE-UV-VIS Analysis	HPLC-DAD Analysis
SAMPLE 1 (L-arginine)	788.85	Measured mass (mg/g sample)	848.2 ± 21.7	877.7 ± 31.7	879.0 ± 16.3
		RSD (%)	2.6	3.6	1.9
		Disagreement from * (%)	7.5	11. 3	11.4
SAMPLE 2 (L-arginine)	333.33	Measured mass (mg/g sample)	341.8 ± 4.2	329.3 ± 22.3	366.7 ± 14.7
		RSD (%)	1.2	6.8	4.0
		Disagreement from * (%)	2.6	−1.1	10.0
SAMPLE 3 (L-ornithine)	742.60	Measured mass (mg/g sample)	795.7 ± 32.7	834.6 ± 49.1	761.9 ± 50.4
		RSD (%)	4.1	5.9	6.6
		Disagreement from * (%)	7.1	12.5	2.62
SAMPLE 4 (L-lysine)	6.65	Measured mass (mg/g sample)	7.0 ± 0.1	6.5 ± 0.52	6.6 ± 0.5
		RSD (%)	1.0	8.0	8.2
		Disagreement from * (%)	5.9	−2.3	−5.6
SAMPLE 5 (L-arginine)	545.03	Measured mass (mg/g sample)	567.1 ± 12.0	580.0 ± 28.5	561.8 ± 23.0
		RSD (%)	2.1	4.9	4.1
		Disagreement from * (%)	4.0	6.4	3.1
SAMPLE 5 (L-ornithine)	293.48	Measured mass (mg/g sample)	304.0 ± 12.0	295.91 ± 15.3	308.83 ± 20.1
		RSD (%)	3.9	4.8	6.5
		Disagreement from * (%)	3.6	9.16 ± 5.2	5.23 ± 6.9

* Number of determinations = 3.

**Table 5:** *t*-test data on t-calculated (t_calc_) values for comparison of the results of measured concentrations of AAs in nutritional supplements obtained with ME-C^4^D, CE-UV-VIS, and HPLC-DAD methods.

		ME-C^4^D and CE-UV-VIS	ME-C^4^D and HPLC-DAD	CE-UV-VIS and HPLC-DAD
SAMPLE 1	L-arginine	0.9581	1.4148	0.1308
SAMPLE 2	L-arginine	0.9581	3.5221	1.9305
SAMPLE 3	L-ornithine	2.8949	3.1048	4.4536
SAMPLE 4	L-lysine	1.8200	1.4462	0.3811
SAMPLE 5	L-arginine	0.6012	0.6740	0.6246
SAMPLE 5	L-ornithine	1.5161	0.3982	3.7558

t-critical (t_crit_) = 4.303, n = 3, α = 0.05.

**Table 6:** The determined concentration of AAs with the ME-C^4^D method and their recovery data.

	Analyte	Native Concentration (μM)	Added Concentration (μM)	Determined Concentration (μM)	Recovery (%)
Recovery test for 20% spiked sample					
Sample 1	L-arginine	48.69 ± 1.24	9.7	58.82 ± 0.41	103.92 ± 4.15
Sample 2	L-arginine	39.25 ± 0.48	7.8	47.37 ± 0.14	104.14 ± 1.76
Sample 3	L-ornithine	39.32 ± 1.61	7.9	46.62 ± 0.11	92.75 ± 1.35
Sample 4	L-lysine	38.64 ± 0.27	7.7	46.07 ± 0.11	96.16 ± 1.51
Sample 5	L-arginine	32.55 ± 0.69	6.51	39.56 ± 0.32	109.54 ± 3.67
Sample 5	L-ornithine	18.02 ± 0.70	3.6	21.73 ± 0.09	102.88 ± 2.08
Recovery test for 40% spiked sample					
Sample 1	L-arginine	48.69 ± 1.24	19.5	67.5 ± 0.38	96.39 ± 1.61
Sample 2	L-arginine	39.25 ± 0.48	15.6	53.75 ± 0.97	93.26 ± 6.19
Sample 3	L-ornithine	39.32 ± 1.61	15.7	54.92 ± 0.36	99.18 ± 2.34
Sample 4	L-lysine	38.64 ± 0.27	15.6	55.4 ± 0.25	108.45 ± 1.65
Sample 5	L-arginine	32.55 ± 0.69	13.02	46.54 ± 0.25	107.41 ± 1.56
Sample 5	L-ornithine	18.02 ± 0.70	7.21	25.81 ± 0.23	107.98 ± 2.62

Number of determinations = 3.

## Data Availability

Data will be made available on request.

## Supplementary Materials

The following supporting information can be downloaded at: https://www.mdpi.com/article/10.3390/molecules29235554/s1. Figure S1. (A) Capillary electrophoresis of Sample 1. CE conditions: 5 × 10^−5^ M CuSO_4_ + 0.05% AcOH electrophoretic buffer (pH = 4.5), hydrodynamic injection at 50 mbar for 5 s and +15 kV separation voltage, the measured current ≈ 10 μA. (B) Capillary electrophoresis of Sample 2. CE conditions: 5 × 10^−5^ M CuSO_4_ + 0.05% AcOH electrophoretic buffer (pH = 4.5), hydrodynamic injection at 50 mbar for 5 s and +15 kV separation voltage, the measured current ≈ 10 μA. (C) Capillary electrophoresis of Sample 3. CE conditions: 5 × 10^−5^ M CuSO_4_ + 0.05% AcOH electrophoretic buffer (pH = 4.5), hydrodynamic injection at 50 mbar for 5 s and +15 kV separation voltage, the measured current ≈ 10 μA. (D) Capillary electrophoresis of Sample 4. CE conditions: 5 × 10^−5^ M CuSO_4_ + 0.05% AcOH electrophoretic buffer (pH = 4.5), hydrodynamic injection at 50 mbar for 5 s, and +15 kV separation voltage, the measured current ≈ 10 μA. (E) Capillary electrophoresis of Sample 5. CE conditions: 5 × 10^−5^ M CuSO_4_ + 0.05% AcOH electrophoretic buffer (pH = 4.5), hydrodynamic injection at 50 mbar for 5 s, and +15 kV separation voltage, the measured current ≈ 10 μA.
