# Supplementary material for: Green Microfluidic Method for Sustainable and High-Speed Analysis of Basic Amino Acids in Nutritional Supplements
Source: Molecules. 2024 Nov 25;29(23):5554. doi: 10.3390/molecules29235554 (PMC11643986; doi:10.3390/molecules29235554)
Supplement: Supplementary file 1 [file molecules-29-05554-s001.zip › molecules-3306889-supplementary.pdf]

Supplementary Materials

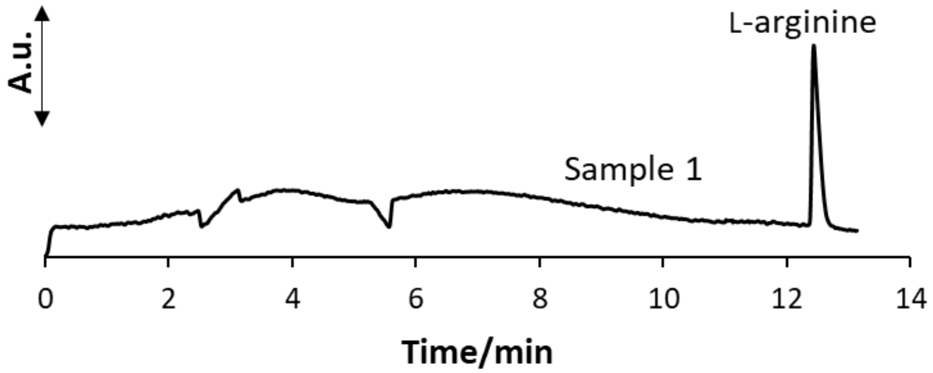

(A)

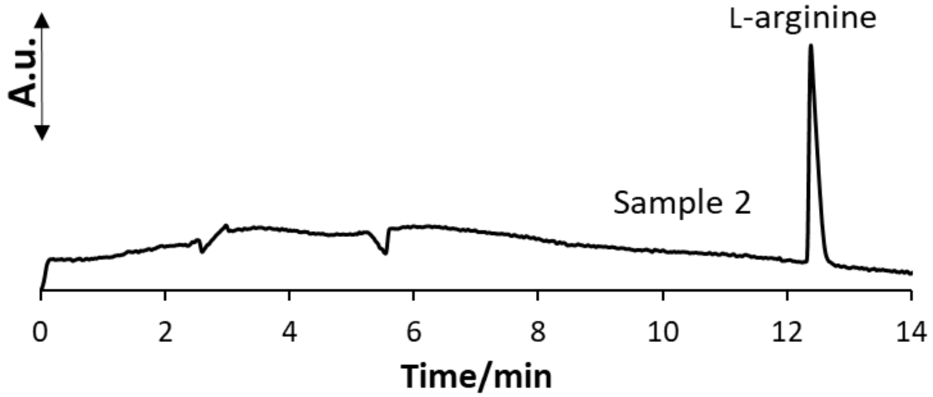

(B)

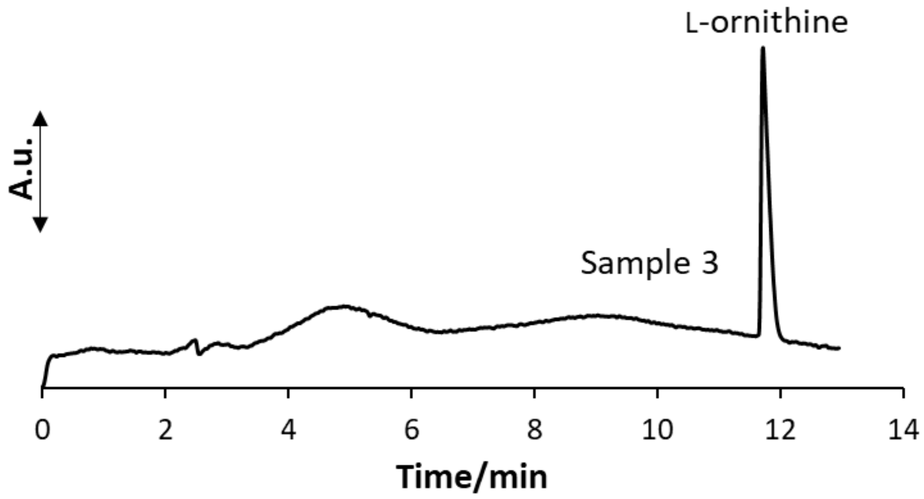
L-ornithine
A.u.
Sample 3
0
2
4
6
8
10
12
14
Time/min

(C)

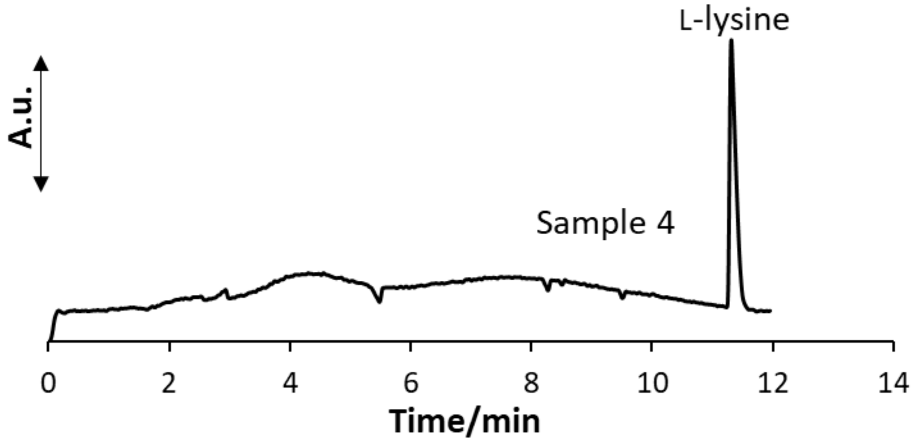
L-lysine
A.u.
Sample 4
0
2
4
6
8
10
12
14
Time/min

(D)

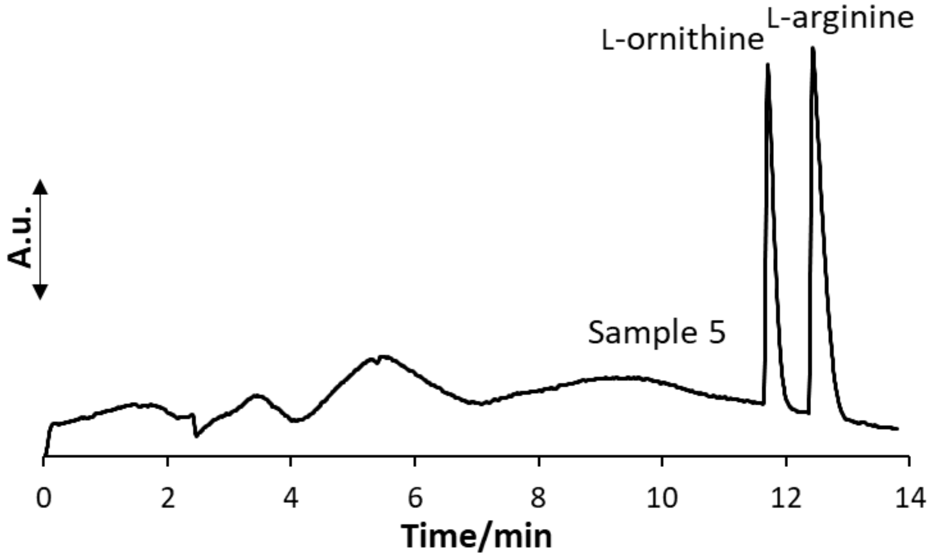

(E)

Figure S1. (A) Capillary electrophoresis of Sample 1. CE conditions: 5 x 10^{-5} M $CuSO_4$ + 0.05% AcOH electrophoretic buffer (pH = 4.5), hydrodynamic injection at 50 mbar for 5 s and +15 kV separation voltage, the measured current ≈ 10 μA**. (B)** Capillary electrophoresis of Sample 2. CE conditions: 5 x 10^{-5} M $CuSO_4$ + 0.05% AcOH electrophoretic buffer (pH = 4.5), hydrodynamic injection at 50 mbar for 5 s and +15 kV separation voltage, the measured current ≈ 10 μA. **(C)** Capillary electrophoresis of Sample 3. CE conditions: 5 x 10^{-5} M $CuSO_4$ + 0.05% AcOH electrophoretic buffer (pH = 4.5), hydrodynamic injection at 50 mbar for 5 s and +15 kV separation voltage, the measured current ≈ 10 μA. **(D)** Capillary electrophoresis of Sample 4. CE conditions: 5 x 10^{-5} M $CuSO_4$ + 0.05% AcOH electrophoretic buffer (pH = 4.5), hydrodynamic injection at 50 mbar for 5 s and +15 kV separation voltage, the measured current ≈ 10 μA. **(E)** Capillary electrophoresis of Sample 5. CE conditions: 5 x 10^{-5} M $CuSO_4$ + 0.05% AcOH electrophoretic buffer (pH = 4.5), hydrodynamic injection at 50 mbar for 5 s and +15 kV separation voltage, the measured current ≈ 10 μA.
